# Modeling Materials Coextrusion in Polymers Additive Manufacturing

**DOI:** 10.3390/ma16020820

**Published:** 2023-01-14

**Authors:** Riccardo Sponchiado, Stefano Rosso, Pierandrea Dal Fabbro, Luca Grigolato, Hamada Elsayed, Enrico Bernardo, Mattia Maltauro, Francesca Uccheddu, Roberto Meneghello, Gianmaria Concheri, Gianpaolo Savio

**Affiliations:** 1 Department of Civil, Environmental, and Architectural Engineering, University of Padova, 35122 Padua, Italy; 2 Department of Industrial Engineering, University of Padova, 35122 Padua, Italy; 3 Refractories, Ceramics and Building Materials Department, National Research Centre, Cairo 12622, Egypt; 4 Department of Management and Engineering, University of Padova, 35122 Padua, Italy

**Keywords:** additive manufacturing, fused deposition modeling, material extrusion, coextrusion, material modeling

## Abstract

Material extrusion additive manufacturing enables us to combine more materials in the same nozzle during the deposition process. This technology, called material coextrusion, generates an expanded range of material properties, which can gradually change in the design domain, ensuring blending or higher bonding/interlocking among the different materials. To exploit the opportunities offered by these technologies, it is necessary to know the behavior of the combined materials according to the materials fractions. In this work, two compatible pairs of materials, namely Polylactic Acid (PLA)-Thermoplastic Polyurethane (TPU) and Acrylonitrile Styrene Acrylate (ASA)-TPU, were investigated by changing the material fractions in the coextrusion process. An original model describing the distribution of the materials is proposed. Based on this, the mechanical properties were investigated by analytical and numerical approaches. The analytical model was developed on the simplified assumption that the coextruded materials are a set of rods, whereas the more realistic numerical model is based on homogenization theory, adopting the finite element analysis of a representative volume element. To verify the deposition model, a specific experimental test was developed, and the modeled material deposition was superimposed and qualitatively compared with the actual microscope images regarding the different deposition directions and material fractions. The analytical and numerical models show similar trends, and it can be assumed that the finite element model has a more realistic behavior due to the higher accuracy of the model description. The elastic moduli obtained by the models was verified in experimental tensile tests. The tensile tests show Young’s moduli of 3425 MPa for PLA, 1812 MPa for ASA, and 162 MPa for TPU. At the intermediate material fraction, the Young’s modulus shows an almost linear trend between PLA and TPU and between ASA and TPU. The ultimate tensile strength values are 63.9 MPa for PLA, 35.7 MPa for ASA, and 63.5 MPa for TPU, whereas at the intermediate material fraction, they assume lower values. In this initial work, the results show a good agreement between models and experiments, providing useful tools for designers and contributing to a new branch in additive manufacturing research.

## 1. Introduction

Recent advancements in additive manufacturing (AM) enable multi-materials processing [1]. From a design standpoint, these innovations provide exceptional opportunities, allowing us to meet several functional requirements in the same component in a single process. Moreover, recent studies show the advantages of multi-materials in achieving the shape memory effect [2,3]. Nevertheless, the literature shows several limits in the design and manufacturing of multi-materials AM components such as compatible materials processing, interface behavior, and mechanical properties [4,5,6].

During the product design, a variation in the properties usually occurred by separating the product into parts with different homogeneous materials. The same variation can be obtained by locally changing the composition to achieve tailored characteristics. Products with a heterogeneous composition can have several delimited areas associated with different functions or a gradual variation in the material fractions among different regions. The latter ones are called functionally graded materials (FGM) and Functionally Graded Additive Manufacturing (FGAM) when one is referring to their AM production [4].

Although FGAM has great potential, its adoption is still limited due to the lack of knowledge mainly related to three bottlenecks: processability, indeed, the materials that can be simultaneously manufactured, and their relevant process parameters are often unknown, and the features of Computer-Aided Manufacturing (CAM) software rarely allow us to process virtual models of FGM; properties, i.e., only few combinations of materials were investigated from a functional point of view; design tools, which means that Computer-Aided Design (CAD) software does not support geometric modeling, analysis, and data exchange for FGM and, in general, for non-homogeneous solids. In this scenario, new CAD/CAM/CAE AM software should be able to optimize the allocation of material in a logical distribution during the generation of the FGM model [7].

Nevertheless, multi-material AM technologies are available in a variety of prosumer and industrial technologies used for FGAM. Among these, due to their diffusion and range of available materials, Fused Deposition Modeling (FDM), also known as Fused Filament Fabrication (FFF) [8], is the material extrusion (MEX) technology adopted in this study.

Due to the simplicity of assembly and the wide range of materials available, FDM has become a widespread technology since the Stratasys’s patent decay [9]. The functioning of the FDM consists of the extrusion of a thermoplastic material, which is supplied as a filament, commonly with a diameter of 1.75 mm. In addition to the common styrenic polymers (Acrylonitrile Butadiene Styrene—ABS; Acrylonitrile Styrene Acrylate—ASA), polyesters (Polyethylene Terephthalate—PET; Polybutylene Terephthalate—PBT), naturally derived polymers (Polylactic Acid—PLA), and polyamides (PA), other materials are available on the market which extend the range of obtainable properties [10]. Some examples of technical polymers are elastomers and polymers loaded with a variety of fillers (carbon, metals, minerals, pigments, and nanomaterials, etc.) that allow us to obtain various properties such as electrical and thermal conductivity, resistance abrasion, and color variations. Moreover, some companies (e.g., 3DXTech [11]) have introduced technopolymers onto the market: Polyetherimide (PEI), Polyetheretherketone (PEEK), and Polyphenylene Sulfide (PPS) are some of the materials that promise metal-replaceable performances. One way to increase the range of properties in the same component is to use graded materials fractions in a single AM process.

When we are dealing with FDM multi-materials, in the most common of cases, the printing head carriage has two or more identical extruders, each of which processes a different material independently [12]. Alternatively, multiple independent extruders can move on an independent head carriage. Another way to add multi-material capabilities to an FDM printer is represented by the multi material units from Prusa3d and Mosaic. Those are standalone systems that can support several filament feeds and combine them to work with most desktop machines. FGMs in MEX can also be achieved by flowing multiple materials using a single nozzle. An example comes from the RepRap project with the diamond hotend [13]. The hotend system features three independent filament lines, which are controlled according to an established fractional composition, and the combined materials come out of the same nozzle. This type of printing head is often named a mixing nozzle. However, due to the high viscosity, resulting in a laminar flow, the materials come out of the nozzle side by side in a process called coextrusion. Garland and Fadel highlighted the challenges in the coextrusion of FGMs, proposing some solutions regarding the design and the software associated with the process planning, based on a discretized gradient [14]. Due to the printing ease and the difference between the elastic moduli, PLA and Nylon were selected for the experiments, demonstrating the feasibility of the production of FGMs by FDM technologies.

As an alternative to coextrusion, materials can be mixed to obtain a blend by passive or active approaches. To obtain color mixing, thermo-fluid dynamics phenomena inside a custom nozzle have been studied in depth by Han et al. [15]. They designed and manufactured a mixing nozzle and simulated the behavior of the melted material inside the extrusion chamber, searching for the most suitable temperature parameters at each extrusion speed to blend the materials. Other research groups have tried to improve the mixing of filaments inside the extruder working on the hardware: Khondoker et al. [16] proposed an approach based on static mixing, whereas Kennedy and Christ [17] worked on dynamic mixing. In the first case [16], the design and characterization of a bi-extruder with a static intermixer was proposed, which can interlock two thermoplastics of different natures, regardless of their miscibility. The components fabricated with this extruder showed a better cohesion between the layers, which reduces the delamination problems. Moreover, the reduction of the extrusion chamber size also reduces the delay between one composition and another one. In general, the use of an intermixer could improve the strength and aesthetic homogeneity when compared to those which were achieve using a coextrusion. Kennedy and Christ [17] proposed a process named “in situ blending”. The extruder was designed to be adaptable to common low-cost machines, and it contains a drill bit that actively mixes the filaments inside the extrusion chamber using different types of bits with an adjustable speed. Using this extruder, the authors produced specimens with blended PLA and Thermoplastic Polyurethane (TPU) or PLA and Nylon. The result, in general, shows how the blending process, which previously required several different steps, can now be carried out in a single step with this extruder, thereby raising the range of possibilities obtainable with a common 3D printer.

This works aims to lay the foundations to understand the FDM coextrusion process, identifying models of material distribution from a geometrical point of view, which is the base used to describe mechanical properties. Based on a simplified deposition model, the couplings of PLA-TPU and ASA-TPU were analytically, numerically, and experimentally investigated by elastic models, the Representative Volume Element (RVE), and tensile tests.

## 2. Materials and Methods

### 2.1. Material Coextrusion

**Figure 1:**
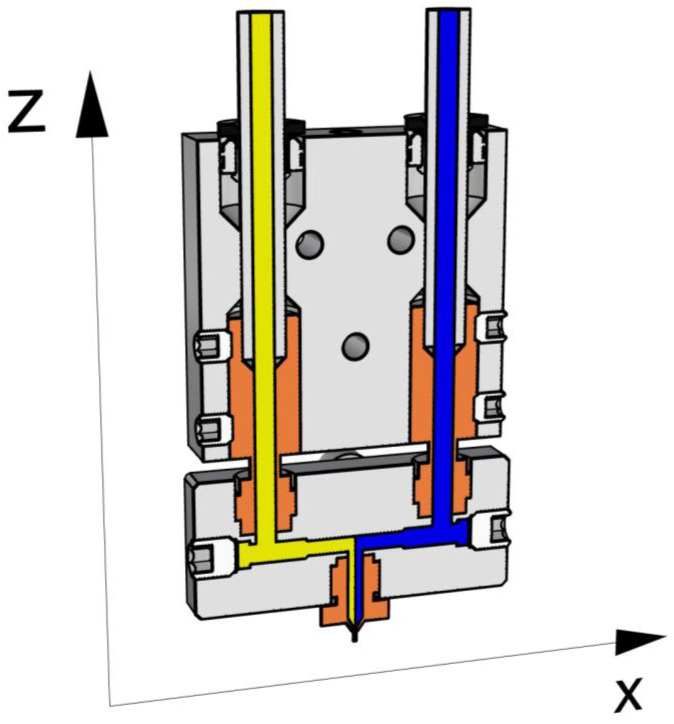
Cyclops hotend representation. In the lower portion (heating block), it is possible to see that both materials converge in the same nozzle (CAD model adapted from GrabCAD [23]).

The FDM technology adopted in this study is a customized Prusa I3 by Geeetech [18], onto which a Rumba motherboard [19] equipped with an adapted Marlin firmware [20] and a Cyclops hotend [21] were mounted. The materials, pushed by a Bondtech BMG dual drive extruder [22], reach the hotend through 2 bowden tubes. A diamond hotend [13] was also tested and achieved similar results. The combined materials simultaneously flowed through the same nozzle, as shown in Figure 1.

According to the computer numerical control programming language (G-code), the required fractions of each material were obtained by simultaneously controlling the amount of material pushed by each extruder.

Adopting the Marlin firmware, the material percentage was coded into the G-code by the commands, M163 and M164. The former one set the mutual amount of each material, whereas the second latter one assigned the mixing to a virtual extruder [24], such as:

M163 S0 P0.75

M163 S1 P0.25

M164 S3

which means that the tool number 3 was set to be 75% of the material in the left channel and 25% of the material in the right channel. To obtain the needed tool, it was necessary introducing the line T*n* in the right position of the G-code listing, where *n* is the number of the tool (3 in the example, i.e., T3) [25]. To use the commands M163 and M164, the Marlin configuration file (Configuration h) must be modified, thereby enabling the mixing extruder [26].

### 2.2. Materials and Process Parameters

The PLA-TPU and ASA-TPU pairs of materials were studied. The choice of these pairs allows us to obtain a wide range of properties, while maintaining rather similar process parameters. In detail, the materials used in this research are PLA Extrafill, ASA Extrafill, and Flexfill TPU 98A (hardness 98 Shore A), which are 3 thermoplastic filaments produced by Fillamentum [27] (Table 1). All of the filaments were supplied in spools in a standard 1.75 mm diameter.

PLA is the most widely used thermoplastic filament for FDM. PLA is a rigid and brittle filament that is easy to use, and it is biodegradable in industrial composting systems. While ASA has similar properties to ABS, it is more eco-friendly and has better UV resistance. TPU is a semi-flexible material that offers high tensile strength and high elongation at break values.

Table 2 summarizes the process parameters used in the specimen manufacturing. The bed temperature is a parameter that facilitates the adhesion between the build plate and the manufacturing part. For ASA-TPU, 80 °C was selected as an intermediate value between the ranges suggested by the supplier for each material, allowing for an appropriate adhesion to occur. The printing speed assumes the same value for both the infill and perimeters to avoid material flow variations that can compromise the actual percentage of the material extruded [34].

### 2.3. Models

#### 2.3.1. Deposition Model

**Figure 2:**
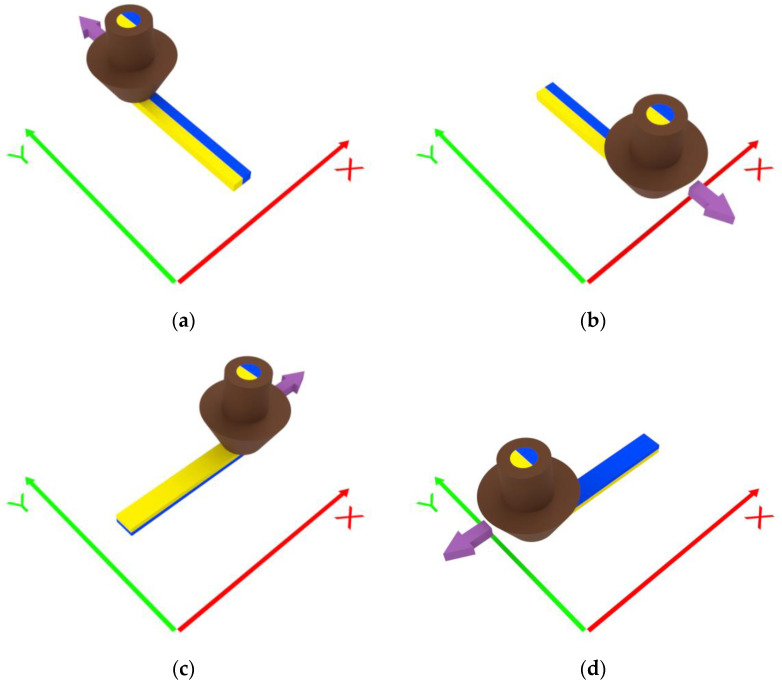
Distribution of the materials deposited depending on the nozzle movement: (**a**) y-positive, (**b**) y-negative, (**c**) x-positive, and (**d**) x-negative directions.

The selected pairs of materials do not mix, instead, they stand side by side in a reciprocal position that depends on the nozzle path. Considering the extruder that was mounted, as shown in Figure 1, due to the hotend configuration, the coextruded materials flow in the nozzle, sharing an interface at a plane that is parallel to the yz-plane. Consequently, when the nozzle moves along the y-axis, the material in the left channel is deposited on the left, while the material flowing in the right channel is deposited on the right (Figure 2a,b). Otherwise, when the nozzle moves along the x-positive direction, the material on the right is deposited below the material on the left (Figure 2c), whereas, when the nozzle moves along the x-negative direction the material flowing on the left is deposited under the material on the right (Figure 2d).

**Figure 3:**
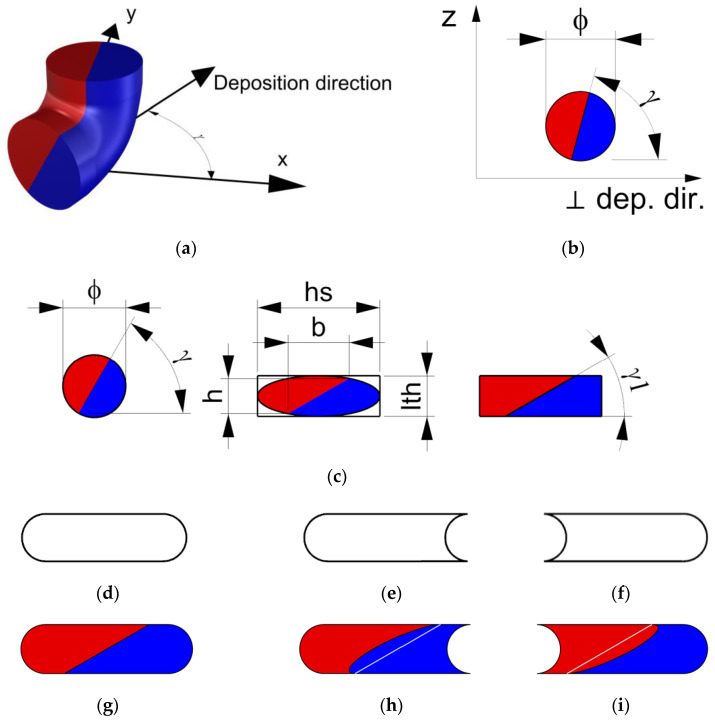
(**a**,**b**) Distribution of the coextruded materials from the nozzle to the deposition plane, considering the deposition direction (**a**), and initial section of the deposited material in a plane perpendicular to the deposition direction (**b**). (**c**) Adaptation of the extruded material to a rectangle. (**d**–**f**) Sections of the deposited material: (**d**) without any adjacent material, (**e**) with previous material deposited on the right, (**f**) and with previous materials deposited on the left. (**g**–**i**) Model of the coextruded material considering a rounded rectangle shape and the previously deposed material for *γ* = 60°: (**g**) without any adjacent material, (**h**) with previous material deposited on the right, (**i**) and with previous materials deposited on the left.

In a generic nozzle path, moving from the vertical axis (nozzle axis, z-axis) to the deposition plane (xy-plane), the coextruded materials rotate by 90° around an axis that is perpendicular to the deposition direction in the deposition plane (⊥ dep. dir. in the xy-plane), as shown in Figure 3a,b. Consequently, for the angle named *γ* between the x-axis and the deposition direction, the line separating the deposited coextruded materials has the same angle *γ*.

During the deposition, the shape of the extruded material changes from a circle with a diameter ϕ at the nozzle to a new shape that can be approximated by a rectangle or a rounded rectangle when the material is solidified [25]. This rectangle has a base equal to the hatching space (hs, distance between 2 adjacent nozzle paths) and a height equal to the layer thickness (lth).

As a first approximation, to adapt the extruded circle to the rectangle, the circle and the materials separation line are scaled to an ellipse inscribed in the rectangle with the axes that are proportional to the rectangle sides (Figure 3c). So, the scale factor along the vertical axis will be assumed to be equal to lth/ϕ, whereas the scale factor in the horizontal direction will be assumed to be equal to hs/ϕ. Consequently, the horizontal (⊥ dep. dir.) and vertical (z-axis) components of the separation line can be calculated as

(1) h=(lth/ϕ)·(ϕ·sin(γ))

(2) b=(hs/ϕ)·(ϕ·cos(γ)),

and the new inclination of the interface between the materials *γ*1 is defined by the equation:

(3) γ1=arctan(h/b)=arctan((lth·sin(γ))/(hs·cos(γ)))=arctan(tan(γ)·lth/hs)

Different fractions of materials flowing from the nozzle can be modeled by simply moving the separation line to the right or left to reflect the materials fraction on the area of each material.

A more realistic model should consider the presence of an adjacent deposited stripe. As mentioned, the deposited material can be modeled as a rounded rectangle if there is no other adjacent deposited material in the same layer (Figure 3d). In the case of the previously deposited material on the right, the new stripe assumes a shape that is similar to that in Figure 3e, whereas if there is material on the left, the stripe assumes the shape in Figure 3f.

After introducing the shape modification of Figure 3d–f into the model of Figure 3c, the separation line can be warped, as in Figure 3, by translating the separation line point by point.

#### 2.3.2. Elastic Modulus Analytical Model

**Figure 4:**
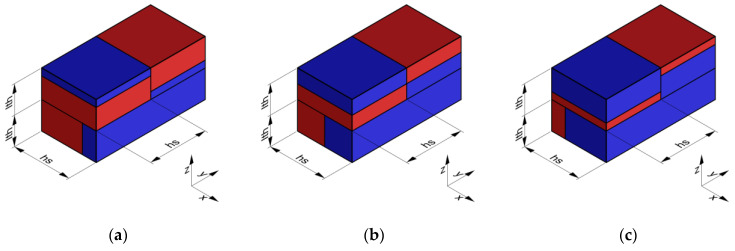

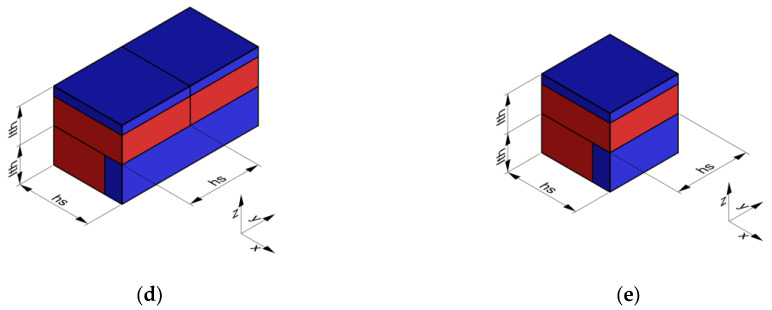
Volume element of deposited material: the first layer is deposited in the y-direction, whereas the second layer is deposited in the x-direction. Blue represents a generic “A material”, and red represents a generic “B material”. In (**a**), the volume element is made up of 25% of the “A material” and 75% of the “B material”, in (**b**), there is 50% of “A” and 50% of “B”, and in (**c**), there is 75% of “A” and 25% of “B”. Simplified model for elastic modulus estimation: (**d**) flipped material extruded along the x-positive direction; (**e**) reduced length.

Under the initial assumption that the nozzle moves along the x-axis in a layer and along the y-axis in the next one and that the deposited material has a rectangular section, a unitary volume element can be described, as in Figure 4a–c, for the different materials’ fractions. The material behaves as a composite which shows different mechanical properties along the x-, y-, and z-axes. To establish the range of the elastic properties as a function of the material fraction, each portion of the volume element was initially modeled as a spring.

To simplify the model, the layer deposited along the x-positive direction was flipped, as shown in Figure 4, and the interaction among the layers and between the adjacent stripes was neglected.

For a rod of length *L*, width *b*, and thickness *t*, the stiffness *k* is defined as

(4) k=E·b·t/L,

where *E* is the elastic modulus of the material. In the proposed models, the stiffness of each element depends on the fraction of “A material” *f*, the fraction of “B material” 1-*f*, *hs*, *lth*, the elastic modulus of “A” *E_A_*, and the elastic modulus of “B” *E_B_*.

To combine 2 or more springs and obtain the resulting stiffness, it is sufficient to apply the relations for a parallel and series combination, which are, respectively:

(5) Kp=k1+k2+…+kn,

(6) 1/ks=1/k1+1/k2+…+1/kn

Based on the above-mentioned assumptions, Table 3 shows the resultant spring models along the x-, y-, and z-axes, where the deposition direction is identified by the numbers 1 (y-direction) and 2 (x-direction).

#### 2.3.3. Mechanical Properties by Homogenization

Numerical simulations were performed to predict the mechanical properties of the combined materials according to the materials fractions and the initial materials properties. As in similar studies [35,36,37], the homogenization method was adopted. Starting from a Representative Volume Element (RVE), i.e., the smallest volume that repeats in the design space, it is possible to define the homogenized material mechanical properties. In the hypothesis of an orthotropic material, these properties are the elastic moduli *E_x_*, *E_y_*, and *E_z_*, the shear moduli *G_xy_*, *G_yz_*, and *G_zx_*, and Poisson’s ratios *ν_xy_*, *ν_xz_*, and *ν_yz_*, which are necessary to define the stiffness matrix *K* of the homogenized material [38]:

(7) $$\left[K\right]=\left[\begin{array}{cccccc} \frac{1}{E_{x}} & -\frac{v_{yx}}{E_{y}} & -\frac{v_{zx}}{E_{z}} & 0 & 0 & 0\\ -\frac{v_{xy}}{E_{x}} & \frac{1}{E_{y}} & -\frac{v_{zy}}{E_{z}} & 0 & 0 & 0\\ -\frac{v_{xz}}{E_{x}} & -\frac{v_{yz}}{E_{y}} & \frac{1}{E_{z}} & 0 & 0 & 0\\ 0 & 0 & 0 & \frac{1}{G_{xy}} & 0 & 0\\ 0 & 0 & 0 & 0 & \frac{1}{G_{yz}} & 0\\ 0 & 0 & 0 & 0 & 0 & \frac{1}{G_{zx}} \end{array}\right]$$

where:

(8) $$\frac{v_{yx}}{E_{y}}=\frac{v_{xy}}{E_{x}},\;\frac{v_{zx}}{E_{z}}=\frac{v_{xz}}{E_{x}},\;\frac{v_{zy}}{E_{z}}=\frac{v_{yz}}{E_{y}}$$

**Figure 5:**
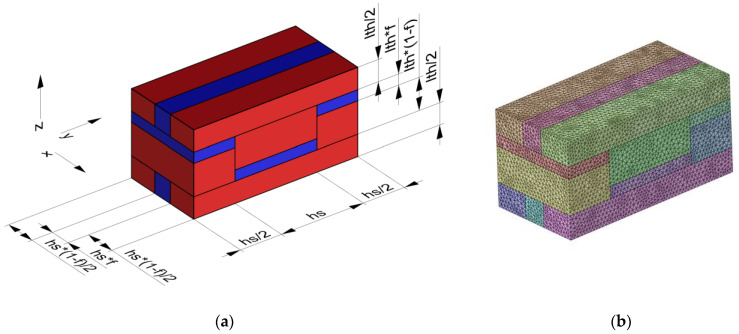
(**a**) RVE dimensions according to the material fraction f. (**b**) Mesh adopted in the simulation.

Linear statical analyses were performed in Ansys Mechanical using the Material Designer tool, and the Representative Volume Element (RVE) was modeled in Ansys Spaceclaim for 7 levels of the material fraction in steps of 0.125. Figure 5a shows the dimensions of the RVE according to the material fraction f. The RVE is obtained from Figure 4a, moving the volume element by a quarter along the x-, y-, and z-axes to achieve coincident topologies on the opposite faces in order to share the same mesh nodes at the interfaces, which is needed for the application of periodic meshing and periodic boundary conditions (PBC). Two pairs of materials were studied (PLA-TPU and ASA-TPU). The bulk elastic moduli were derived from the tensile tests, whereas the Poisson’s ratio was assumed from the literature (see Table 1). In order to predict the effective properties of the heterogeneous materials, a boundary value problem may be defined on an RVE with PBC; many numerical studies show that PBC is the most efficient one in terms of the convergence rate [35,37,39,40].

After a preliminary convergence study, the mesh size was set to 0.02 mm, obtaining the mesh shown in Figure 5b, with a number of tetrahedral elements as follows (average 167,000 elements), which are, according to the level of material fraction:

- 168,324 elements for 12.5%;
- 166,174 elements for 25.0%;
- 173,352 elements for 37.5%:
- 172,188 elements for 50.0%;
- 156,566 elements for 62.5%;
- 165,834 elements for 75.0%;
- 167,936 elements for 87.5%.

### 2.4. Experimental Characterization

#### 2.4.1. Deposition

**Figure 6:**
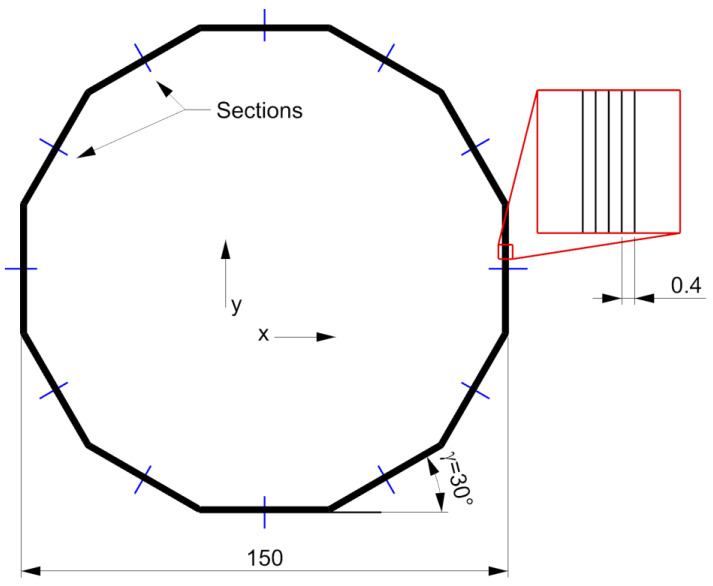
Deposited section studied.

To evaluate the actual interface between the deposited materials, a dedicated G-code was developed. The G-code was computed from a curve that describes the nozzle path in which a regular dodecagon of 150 mm is repeated 5 times in the same layer by an offset distance *hs* = 0.4 mm, and it is repeated for 5 layers (*lth* = 0.2 mm). The nozzle moves counterclockwise from the outside to the inside (Figure 6). Each side of the dodecagon was cut in half, and the section was analyzed using an optical microscope (Leica MZ 7.5 equipped with an IC 90 E camera). To avoid boundary effects, only the central element of each section was studied. Unlike other shapes, the polygon allows one to better identify the actual deposition direction angle *γ*. Three pairs of materials were considered: PLA-PLA, PLA-TPU, and ASA-TPU.

Moreover, images were acquired by scanning electron microscopy (SEM, FEI Quanta 200 ESEM, Eindhoven, Netherlands) to study the interface among the different materials and between the layers.

#### 2.4.2. Mechanical Properties

The mechanical tensile properties of the FDM-printed specimens depend on the direction of the deposition of the filament. So, it is useful to consider different deposition patterns to evaluate the mechanical properties both in the direction parallel to the deposition path and in the perpendicular direction to measure intra-layer adhesion. As a preliminary investigation, to limit the tests and outline an initial picture, we operated in an intermediate condition, aligning the coextruded samples along the diagonal of the building plate and adopting a deposition direction perpendicular to the edges of the samples (i.e., *γ* = ±45° and *γ* = ±135°).

**Figure 7:**
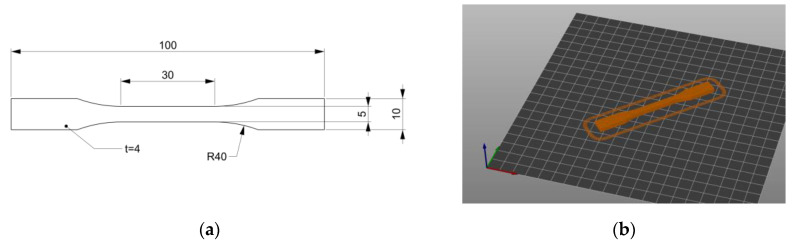
Tensile specimen, 1BA type, according to ISO 527 standard: (**a**) dimensions and (**b**) manufacturing orientation with respect to the building plate.

According to the ISO/ASTM DIS 52,927 international standard (under development at the time of writing) [41], the tensile strength of the additively manufactured polymer parts can be tested following the ISO 527 standard [42]. Figure 7 shows the dimensions of the type 1BA test specimens and the manufacturing orientation with respect to the building plate. More, according to the ISO/ASTM 52,903 international standard [43], three samples for each pair of materials (PLA-TPU and ASA-TPU) and the material fraction (0, 0.25, 0.5, 0.75, and 1) were produced for a total of 27 specimens.

Since very high elongations are expected, especially for the TPU specimens, two types of test apparatus were adopted. The first one focused on the linear zone to obtain the elastic modulus, whereas the second one aimed to obtain the complete stress–strain curve of the specimens up to the point of failure. The tests were executed as follows:

- For the determination of the elastic modulus, the tests were performed on a MTS Electrodynamic Test Systems Acumen 3 equipped with a 3 kN load cell and a MTS 634.31 F extensometer. A 25 mm gauge length was used, and the test rate was 0.25 mm/min.
- For the characterization of the specimens up to failure, a Galdabini SUN 2500 equipped with a 25 kN load cell and a Galdabini PLAST extensometer was used. The test rate was 10 mm/min.

The samples were tested for the elastic modulus (E), also referred to as Young’s modulus, the ultimate tensile strength (UTS), and the maximum strain at failure (ε_max_).

## 3. Results and Discussions

Table 4 summarized the investigated cases. The validation of the deposition model was obtained by the superimposition of the microscope image with the expected results. The comparison of the mechanical properties computed by the models and experiments was performed on the elastic modulus.

**Figure 8:**
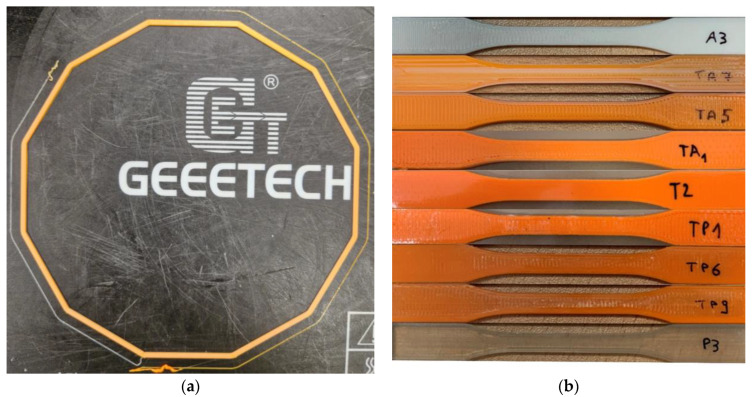
Additively manufactured samples. (**a**) Deposition test (dodecagon shape) ASA-TPU. (**b**) Tensile test samples from top to bottom: 100% ASA; 75% ASA and 25% TPU; 50% ASA and 50% TPU; 25% ASA and 75% TPU; 100% TPU; 25% PLA and 75% TPU; 50% PLA and 50% TPU; 75% PLA and 25% TPU; 100% PLA.

Figure 8 shows some of the manufactured samples; the regular dodecagon used to evaluate the interface between the deposited materials is shown in Figure 8a, whereas Figure 8b shows one tensile test specimen for each material mixture.

**Figure 9:**
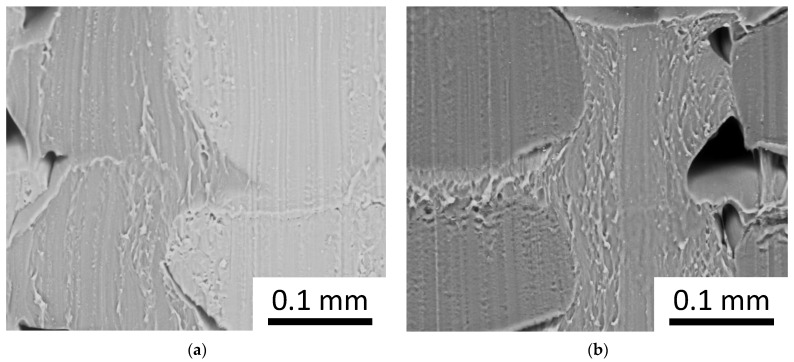
SEM images of the materials interfaces for (**a**) PLA-TPU (γ = 90°) and (**b**) ASA-TPU (γ = 90°); material fraction: 50%.

Figure 9 shows the SEM images of a section of the dodecagon with a 0.5 material fraction. In both of the mixes, i.e., PLA-TPU and ASA-TPU, the coextruded stripes show a clear separation between the materials, i.e., the materials did not mix, and they consolidated together with an appropriately strong adhesion. Moving to the interface between the stripes, in the same or in different layers, a good adhesion between the materials can be observed, even if a few pores appear, which are typical of the FDM process [44,45]. These defects can be mitigated by the tuning process parameters such as the raster angle, build orientation, flow, and temperature [46,47,48]. Moreover, the different melting temperature of the materials leads to a different viscosity, and more fluid material allows for a better flow in the nozzle and leads to a better coverage of the pores. Other aspects that influence material adhesion are related to adsorption, diffusion, and electrostatic phenomena [49]. In the coextrusion process, the more compliant material reduces the product defects and increases the manufacturability; indeed, the TPU compliance and adhesion compensate for the solidification shrinkage of the other material, especially in the case of ASA, reducing the delamination among the layers and increasing the strength of the adhesion to the printer bed surface.

### 3.1. Materials Deposition

**Figure 10:**
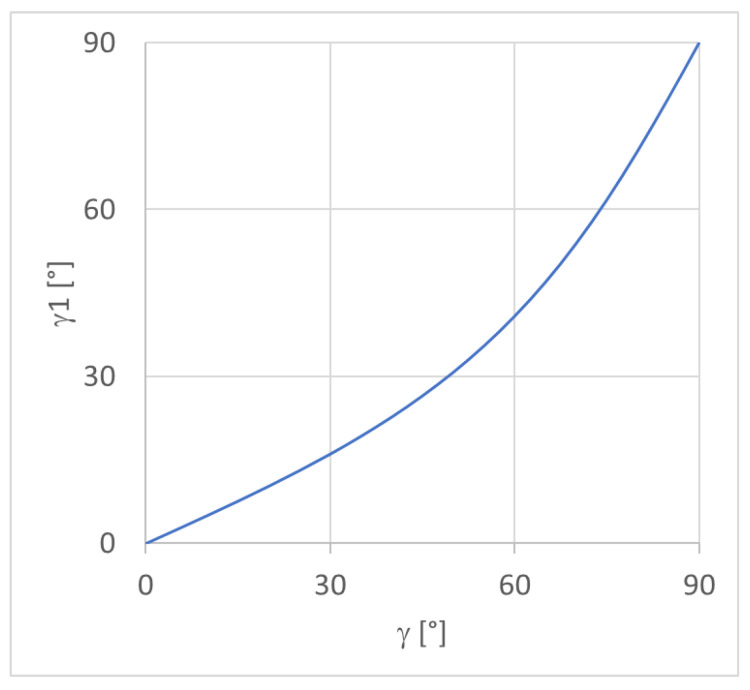
*γ*1 vs. *γ* for *lth* = 0.2 mm and *hs* = 0.4 mm.

Using the adopted process parameters, i.e., *lth* = 0.2 mm and *hs* = 0.4 mm, the inclination of the interface between the materials *γ*1 (Figure 3c) is represented in Figure 10, which was acquired according to the deposition direction *γ* and Equation (3). The trend is not linear, and the inclination of the interface is always lower than the deposition angle.

**Figure 11:**
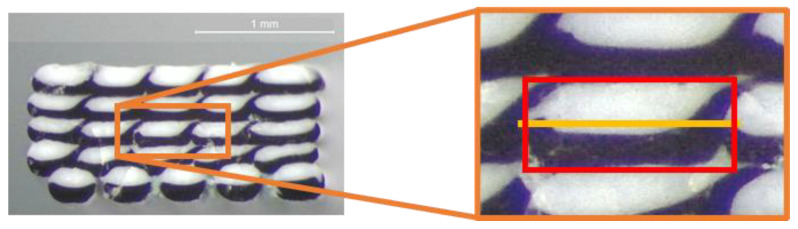
Analysis of the dodecagon section and deposition model superimposition.

To evaluate the actual interface between the coextruded materials and avoid boundary effects, the central stripe of each dodecagon section, representing the 3rd stripe of the 3rd layer, was extracted and superimposed onto the rectangular deposition model presented in Figure 3c, together with the angles of Figure 10 (Equation (3)), as shown in Figure 11. The rectangular deposition model was selected to reflect the condition of the analytical model and the RVE analyses.

**Figure 12:**
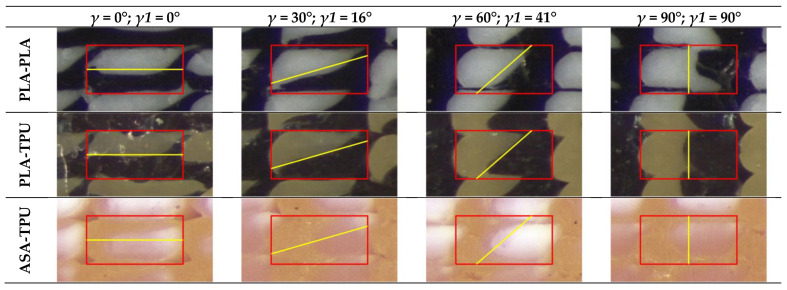
Actual cross section of the material deposited and rectangular model superimposition for the pairs of materials PLA-PLA 50–50%; PLA-TPU 50–50%; ASA-TPU 50–50%. The rectangular model is highlighted in red, whereas the inclination of the interface is highlighted in yellow.

Figure 12 shows the actual cross section of the material deposited in the dodecagon, which was superimposed onto the rectangular model for the three pairs of materials, PLA-PLA, PLA-TPU, and ASA-TPU, at 50% of the material fraction. Due to the periodic behavior, only the results in the range of 0–90° are presented. The yellow lines represent the inclination of the interface between the materials *γ*1 according to Equation (3) and Figure 10, while the red rectangle represents the rectangular deposition model (Figure 3). As observed in the SEM images, a clear interface between the coextruded materials appears both inside each stripe and between the stripes. It can be observed that the proposed model accurately represents the actual angle formed between the two coextruded materials especially in the pairs PLA-PLA and PLA-TPU. ASA-TPU slightly differs from the model; in this case, there are bigger deviations such as non-linear “shapes” in the material interfaces, such as squeezing or warping in the center or alterations towards the corners. The deviations from the model can be due to the differences in the materials’ rheological properties according to the temperature, such as surface tension, viscosity, diffusion, the hotend dynamics [45], the deposition order of the pattern, and the polymers’ density. For example, the upper right corner of every section forms a hook-like shape involving the downer material due to the deposition order so that the deposited material “anchors” to the previously deposited one. Moreover, porosities can be appreciated at the four corners among the stripes that are typical of the FDM process, and they can be removed, as previously discussed, by tuning the process parameters.

**Figure 13:**
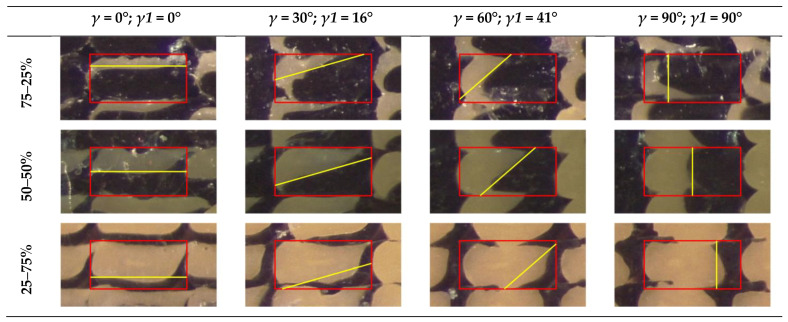
Actual cross section of the material deposited and rectangular model superimposition for the couple PLA-TPU for different material fractions. The rectangular model is highlighted in red, whereas the inclination of the interface is highlighted in yellow.

Figure 13 shows the actual cross section of the deposited material, which was superimposed to the rectangular model of PLA-TPU for three material fractions: 75–25%; 50–50%; 25–75%. The yellow lines are translated to reflect the material fraction in the rectangular model (red line), as proposed in Section 2.3. Considerations that are similar to the previous case can be made, and it is possible to observe that the translation related to the material fraction is effective. Moreover, the interface between the coextruded material is not perfectly straight, and it is more similar to the model based on the rounded rectangle as shown in Figure 3. On the other hand, this first approximation can be considered to be adequate for the analytical model and for the RVE simulations.

### 3.2. Mechanical Tests

**Figure 14:**
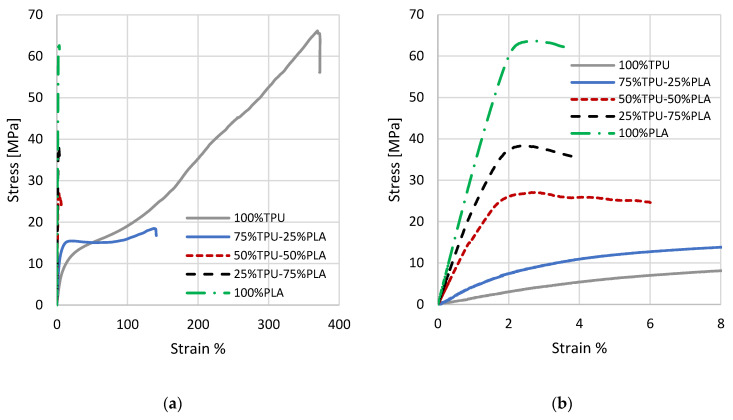
(**a**) Stress–strain curves for the different PLA-TPU mixes. (**b**) Enlargement of the stress–strain curves at low deformation percentages.

Figure 14 a shows representative stress–strain curves for the different mixing percentages of the PLA-TPU samples, with an enlargement in the first section of the curve (Figure 14b) to better appreciate the different elastic behaviors.

**Figure 15:**
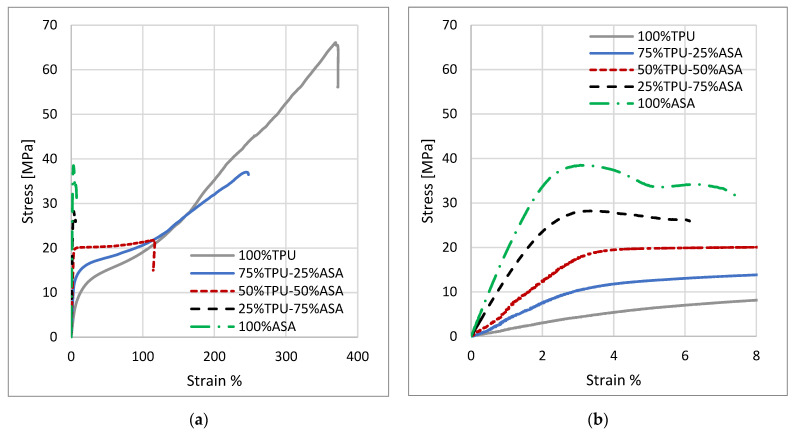
(**a**) Stress–strain curves for the different ASA-TPU mixes. (**b**) Enlargement of the stress–strain curves at low deformation percentages.

Similarly, Figure 15 shows a representative stress–strain curve for each mixture of the ASA-TPU series.

**Figure 16:**
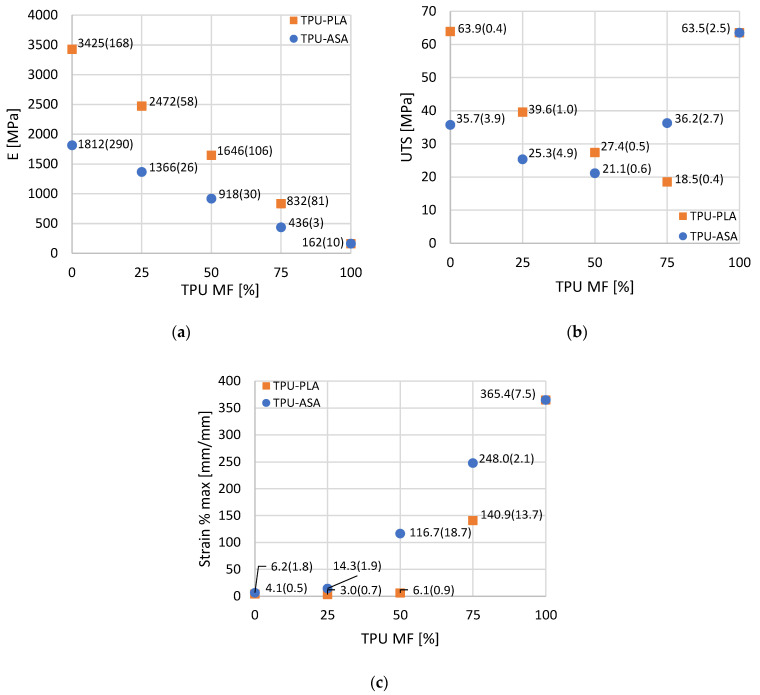
Results of the mechanical tests: (**a**) Young’s modulus, (**b**) Ultimate Tensile Strength, and (**c**) maximum strain.

The results of the analysis of the Young’s modulus, UTS, and maximum strain for the PLA-TPU and ASA-TPU series are summarized in the plots in Figure 16.

The results of the non-mixed materials, i.e., PLA, TPU, and ASA, are in agreement with the data declared by the filament producer [27]. A mismatch was found for the maximum strain of the ASA samples, where the obtained mean value, 6.2%, differs from that of the datasheet, 35%. Nevertheless, similar maximum strain values of 6% can be found in the literature, for instance, Vazquez Martinez et al. [50] obtained comparable results by additively manufacturing ASA specimens by testing several process parameter combinations. Moreover, in general, an agreement is found between the results of the mechanical characterization in the present study with those of other studies in the literature for PLA [51,52,53], ASA [50,54], and TPU [55].

Moving onto the mixed materials, the Young’s modulus at different percentages of TPU (Figure 16a) shows a comparable trend for both the PLA-TPU and the ASA-TPU series: it monotonically decreases, thereby increasing the TPU percentages. Conversely, the strain at failure (Figure 16c) increases at higher TPU percentages, apart from, however, the 75% PLA and 25% TPU mix combination that does not undermine the observed trend due to the close values with the surrounding mix combinations and cannot be considered to be significantly different from the previous data. Furthermore, at up to 50% PLA, the elongation at failure does not increase significantly. A different trend was observed for the UTS (Figure 16b), in which the performance of the material mixes was worse than those of the single materials. In this case, the behavior is not monotonic: starting from the UTS values of pure PLA (63.9 MPa) and ASA (35.7 MPa), the UTS decreases for the intermediate mixes, reaching the lowest value at the 25% PLA and 75% TPU (18.5 MPa) and 50% ASA and 50% TPU (21.1 MPa) combinations, then it increasing again up to the pure TPU value (63.5 MPa). This is due to the higher stiffness of PLA and ASA, which tend to bear the load more than TPU does. Indeed, at the same level of deformation, the stress is higher in the stiffer material, i.e., PLA or ASA, which breaks at a lower value of strain. By increasing the percentages of TPU, the sectional area of PLA and ASA decreases, leading to the sudden failure of the samples. On the contrary, at higher material fractions of TPU, when the PLA and ASA stripes are broken, the UTS is related to the sectional area of TPU, and it consequently increases. Although the maximum UTS is reached when we were using a single material, and the other properties, such as elastic modulus, have a continuous variation according to the MF. This allows one to obtain a wide range of properties that the designer can use in a single component, and it helps to reduce the criticism at the interfaces between the different materials.

A comparison can be made with the work of Arifvianto et al. [56], in which PLA and TPU samples produced by AM were compared to a 50/50 mix, and as in the present study, the mix presented an intermediate Young modulus, whereas the strain at break did not increase by adding TPU. It must be highlighted that in Arifvianto et al.’s study [56], the samples were not obtained by coextrusion, but by adopting a so-called “sandwich structure”, where the PLA and TPU layers were deposited in an alternating manner, and the mechanical properties of the starting materials were different from the ones of the present work. More, the results of the UTS value of the 50% PLA and 50% TPU mix (27.4 MPa) are in the range of the ones obtained by Kennedy and Christ [17] (17 MPa–38 MPa), who tested 50% PLA and 50% TPU blends obtained by “in situ active mixing” and manufactured tensile specimens both parallel and perpendicular to the applied test load. The elastic modulus of the present study for a 50/50 mix of PLA and TPU (1646 MPa) is higher than the ones of Kennedy and Christ (889 MPa maximum); this could be explained by the use of different materials, process parameters, and different material mixing approaches. Another comparison can be made with the work of Rahmatabadi et al. [57]. In the study, compounds of PLA-TPU (90A) at different mixes were obtained by blending granules into a mixer and by further producing a filament; the tensile samples were manufactured by aligning them to the tensile loading direction. Even though the TPU used in the study, TPU 90A, and the one used in the present work, TPU 98A, slightly differ, and the material mixes were obtained adopting two different approaches, i.e., melt mixing method and coextrusion, the UTS values are in good agreement. Indeed, the two UTS trends show a monotonous decrease when the TPU percentage increases. Furthermore, the UTSs of the 50/50 mix of PLA and TPU are similar: 27.3 MPa in Rahmatabadi et al. and 27.4 MPa in the present work. Additionally, the UTS of the 70% PLA and 30% TPU mix in Rahmatabadi et al. (40.9 MPa) is close to that of the 75% PLA and 25% TPU mix obtained in the present study (39.6 MPa).

### 3.3. RVE Analysis, Analytical Model, and Elastic Modulus Comparison

The results of the numerical simulations according to the material fraction are reported in Table 5 for PLA-TPU and in Table 6 for ASA-TPU.

**Figure 17:**
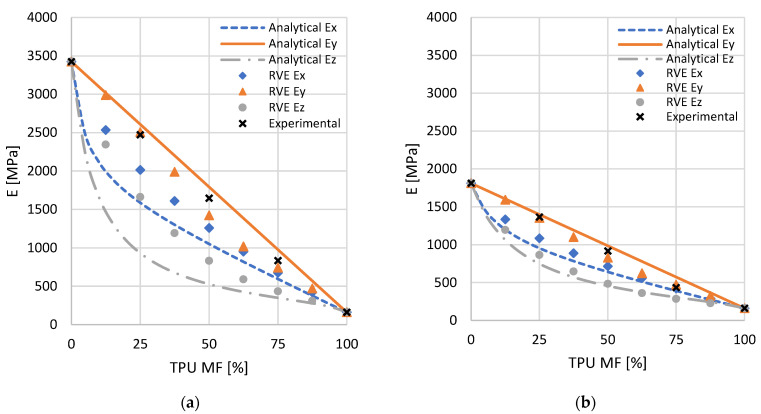
Comparison of the elastic moduli of analytical model, RVE, and tensile tests for the pairs: (**a**) PLA-TPU and (**b**) PLA-TPU.

As expected, both the elastic modulus and the shear modulus monotonically decrease from the 100% PLA or 100% ASA value to the pure TPU value when the fraction of TPU increases. The decreasing E_y_ modulus follows a more linear trend, whereas the decreasing trend of the E_x_ and E_z_ moduli is initially greater (Figure 17). The decreasing trends of the shear moduli are not linear, with G_zx_ being the farthest one from linearity, and with G_xy_ and G_yz_ presenting similar trends. The Poisson’s ratios present a continuous, but not monotonous, trend with the increasing of the percentage of TPU. In terms of the relative trends, the mechanical properties of the two mixes, i.e., PLA-TPU and ASA-TPU, present similar behavior when we are comparing the same parameter.

Figure 17 shows a comparison among the elastic moduli of the analytical model, RVE, and the tensile tests according to the MF. According to Section 2.3.2, the curves of the analytical model were obtained by applying the equations in Table 3 and using the elastic moduli of the pure materials, i.e., PLA, ASA, and TPU, from the results of the mechanical tests presented in the previous section. It is worth noting that the analytical model and RVE show a similar behavior for both of the pairs of materials, where the RVE data show a smaller range. Due to the more advanced model, it can be assumed that the RVE data show more realistic behavior, whereas the analytical model can be considered to be a simplified description that confirms the RVE results. Although the experimental data are obtained under different conditions (e.g., orientations in the building plate and contour patterns for the perimeters), the experimental elastic modulus shows a behavior that is in the range of the models and is close to the linearity. For a more accurate comparison between the finite element models and experimental tests, two specific RVE should be defined: one that is representative of the perimeters and one that is representative of the specific infill deposition orientation. The material properties computed by the RVE should be assigned to the specimen model, which should undergo a simulated traction force for the computation of stress and strain. Moreover, the simulation can include elastoplastic and porosity modeling.

### 3.4. Further Considerations

The proposed models can be useful in the design process of FGM, allowing researcher to establish MF and process parameters according to the functional requirements. Indeed, the model describing the mechanical properties depending on the nozzle path, other process parameters, and the MF can be integrated in a FGM/FGAM design framework [58,59], as the knowledge of the properties of combined materials and their relation to process parameters helps researcher to establish the better products and process configurations. Additionally, this knowledge can be introduced in the iterative design phases, which allows researchers to select the pairs of materials and their distributions. Depending on the continuous or discrete gradient, the proposed models can be integrated into the definition or the material composition function or in the location and size of sub-volume with specific MF and processes parameters.

In the literature, other pairs of materials were considered, such as ABS–TPU, PCL–TPU, PLA/NinjaFlex^®^, and ABS/NinjaFlex^®^ [2,49,60], which could be introduced in the design framework during the material selection phase. Instead of polymers, other materials such as ceramics and metals or a combination of them can be considered by integrating FDM with furnace sintering. To identify the possible compatible pairs, instead of the melting temperature, other aspects should be considered to obtain adhesion between the materials, and consequently, adequate mechanical properties. As highlighted in the literature [2,49,60], the main adhesion mechanisms are related to Van der Waals forces, chemical bonds, wettability, diffusion, and impurities at the boundary. Additionally, the coefficients of thermal expansion play a key role in shape and dimensional accuracy, residual stress, and interface strength. This could lead to deformations of the manufactured parts, which can be reduced by tuning the temperatures and adopting symmetrical material distributions. Moreover, surface roughness and shape interface lead to mechanical adhesion due to microscopic and macroscopic interlocking. Regardless of the adhesion mechanisms, mechanical tests are the most common tools used to establish the interface strength and to confirm adhesion models.

## 4. Conclusions

The capability to combine different material fractions in an additive manufacturing process presents new opportunities in the design of components. To exploit this opportunity, the designer needs models which describe the behavior of the combined materials. This goal was achieved by proposing models which describe the material deposition and mechanical properties and testing two pairs of FDM coextruded materials, i.e., PLA-TPU and ASA-TPU.

The material deposition configuration was studied based on simple geometrical considerations by identifying a model that was superimposed onto the microscope images of the actual deposited material, thereby achieving a good agreement with the experimental tests. While the model can be improved considering the number of phenomena related to the rheological properties of the materials and their variation according to the temperature, the proposed approach is able to explain some of the evidence in an effective way, and it can be considered to be adequate as a basis for analytical and numerical models of the mechanical properties. On the other hand, the process parameters and dedicated slicing procedures should be developed. As an advantage, the selection of adequate pairs of materials can improve the product manufacturability and quality of the components, reducing the delamination and separation from the platform during the fabrication.

Based on the deposition model, an analytical, simplified model of the elastic modulus is proposed and compared to the homogenization approach investigated by a Representative Volume Element in an Ansys environment. The results show that the two approaches are consistent. After tuning the RVE analysis, other deposition conditions should be tested, and the typical morphology of FDM manufacturing, such as a rounded rectangle, and the porosity can be integrated into the volume element.

Experimental tensile tests were performed, and the actual elastic moduli are in the range of the numerical models, according to the MF. The tensile tests show Young’s moduli of 3425 MPa for PLA, 1812 MPa for ASA, and 162 MPa for TPU. At the intermediate material fraction, the Young’s modulus shows an almost linear trend between PLA and TPU and between ASA and TPU. The ultimate tensile strength values are 63.9 MPa for PLA, 35.7 MPa for ASA, and 63.5 MPa for TPU, whereas at intermediate material fraction, they assume lower values. Further tests should be performed to verify the effectiveness of the models, considering, also, different manufacturing directions.

In this preliminary work, the foundations for a methodological approach to modeling FDM coextrusion were laid out, leaving plenty of room for the improvement of the process and the investigation of other materials.

## Figures and Tables

**Table 1:** Main properties of the chosen materials [27].

Properties	PLA Extrafill	ASA Extrafill	Flexfill TPU 98A
Density (g/cm^3^)	1.24	1.07	1.23
Ultimate tensile strength (MPa)	60	40	53.7
Elongation at break (%)	6	35	318
Tensile modulus (MPa)	3600	1726	-
Poisson ratio	0.33 [28,29]	0.38 [30,31]	0.45 [32,33]
Heat distortion temperature (at 0.45 (MPa)) [°C]	55	96	-
Print temperature (range) (°C)	190–210	240–255	220–240
Bed temperature (range) (°C)	55–60	90–105	50–60

**Table 2:** Specimens process parameters.

Properties	PLA-TPU	ASA-TPU
Nozzle temperature (°C)	220	240
Bed temperature (°C)	60	80
Printing speed (mm/s)	30	
Nozzle diameter (mm)	0.4	
Layer thickness (mm)	0.2	
Number of perimeters	3	
Infill density	100%	

**Table 3:** Spring models and resultant elastic modulus along the x, y, and z directions.

Spring Model	Spring Stiffness	Elastic Modulus
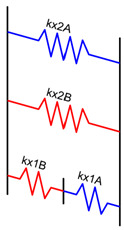	$kx2A=\frac{E_{A}\cdot hs\cdot \left(lth\cdot f\right)}{hs}\;kx2B=\frac{E_{B}\cdot hs\cdot \left(lth\cdot \left(1-f\right)\right)}{hs}$ $kx1B=\frac{E_{B}\cdot hs\cdot lth}{hs\cdot \left(1-f\right)}$ $kx1A=\frac{E_{A}\cdot hs\cdot lth}{hs\cdot f}$	$kx=lth\cdot \left(\frac{E_{A}\cdot E_{B}}{\left(1-f\right)\cdot E_{A}+f\cdot E_{B}}+f\cdot E_{A}+\left(1-f\right)\cdot E_{B}\right)$ $E_{x}=\frac{1}{2}\left(\frac{E_{A}\cdot E_{B}}{\left(1-f\right)\cdot E_{A}+f\cdot E_{B}}+f\cdot E_{A}+\left(1-f\right)\cdot E_{B}\right)$
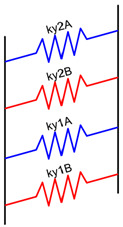	$ky2A=\frac{E_{A}\cdot hs\cdot \left(lth\cdot f\right)}{hs}$ $ky2B=\frac{E_{B}\cdot hs\cdot \left(lth\cdot \left(1-f\right)\right)}{hs}$ $ky1A=\frac{E_{A}\cdot \left(hs\cdot f\right)\cdot lth}{hs}$ $ky1B=\frac{E_{B}\cdot \left(hs\cdot \left(1-f\right)\right)\cdot lth}{hs}$	$ky=2\cdot lth\cdot (f\cdot E_{A}+\left(1-f\right)\cdot E_{B})\;E_{y}=f\cdot E_{A}+\left(1-f\right)\cdot E_{B}$
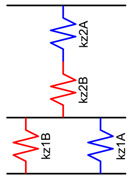	$kz2A=\frac{E_{A}\cdot hs\cdot hs}{lth\cdot f}$ $kz2B=\frac{E_{B}\cdot hs\cdot hs}{lth\cdot \left(1-f\right)}$ $kz1B=\frac{E_{B}\cdot \left(hs\cdot \left(1-f\right)\right)\cdot hs}{lth}$ $kz1A=\frac{E_{A}\cdot \left(hs\cdot f\right)\cdot hs}{lth}$	$\frac{1}{kz}=\frac{lth}{hs^{2}}\cdot \left(\frac{f}{E_{A}}+\frac{\left(1-f\right)}{E_{B}}+\frac{1}{\;f\cdot E_{A}+\left(1-f\right)\cdot E_{B}}\right)$ $E_{z}=\frac{2}{\frac{f}{E_{A}}+\frac{\left(1-f\right)}{E_{B}}+\frac{1}{\;f\cdot E_{A}+\left(1-f\right)\cdot E_{B}}}$

**Table 4:** Summary of the investigated cases.

Studied Feature	Models	Experiments
Deposition	Rectangular model, Equation (3)	Dodecagon, Optical microscope
Adhesion/porosity	/	SEM
Mechanical properties	Analytical model, Table 3	Tensile tests
	Homogenization based on RVE FEM analysis	

**Table 5:** Coefficients of the stiffness matrix, depending on the material fraction, obtained by homogenization of the PLA-TPU RVE.

%TPU (100-%PLA)	0.0%	12.5%	25.0%	37.5%	50.0%	62.5%	75.0%	87.5%	100.0%
E_x_ [MPa]	3425.0	2532.7	2014.3	1609.3	1259.2	949.2	674.3	419.4	161.6
E_y_ [MPa]	3425.0	2988.2	2504.7	1988.5	1422.2	1016.9	739.0	468.3	161.6
E_z_ [MPa]	3425.0	2345.1	1662.0	1190.0	831.1	588.7	434.0	308.6	161.6
G_xy_ [MPa]	1287.6	861.6	674.2	512.0	344.6	214.7	151.5	104.5	55.9
G_yz_ [MPa]	1287.6	800.4	624.6	481.7	318.5	173.0	120.6	88.0	55.9
G_zx_ [MPa]	1287.6	605.1	427.8	306.7	200.1	116.5	84.7	66.2	55.9
ν_xy_	0.330	0.284	0.262	0.244	0.222	0.212	0.227	0.276	0.450
ν_xz_	0.330	0.321	0.352	0.398	0.457	0.516	0.547	0.544	0.450
ν_yz_	0.330	0.352	0.392	0.448	0.523	0.555	0.562	0.538	0.450

**Table 6:** Coefficients of the stiffness matrix, depending on the material fraction, obtained by homogenization of the ASA-TPU RVE.

%TPU (100-%ASA)	0.0%	12.5%	25.0%	37.5%	50.0%	62.5%	75.0%	87.5%	100.0%
E_x_ [MPa]	1812.1	1445.6	1180.2	961.1	768.5	597.3	444.7	303.7	161.6
E_y_ [MPa]	1812.1	1592.7	1353.4	1100.8	833.5	632.5	479.4	329.6	161.6
E_z_ [MPa]	1812.1	1395.3	1065.9	807.1	596.3	448.6	345.1	259.0	161.6
G_xy_ [MPa]	656.6	474.9	378.1	295.8	215.9	154.9	117.5	87.1	55.9
G_yz_ [MPa]	656.6	456.2	362.9	288.5	210.6	139.5	103.3	78.9	55.9
G_zx_ [MPa]	656.6	377.9	277.6	209.0	150.4	103.9	80.1	65.0	55.9
ν_xy_	0.380	0.347	0.327	0.311	0.295	0.290	0.304	0.346	0.450
ν_xz_	0.380	0.373	0.392	0.423	0.462	0.496	0.511	0.501	0.450
ν_yz_	0.380	0.394	0.418	0.454	0.502	0.523	0.520	0.495	0.450

## Data Availability

The data presented in this study are available on request from the corresponding author.
